# Multiparametric transrectal ultrasound for the diagnosis of peripheral zone prostate cancer and clinically significant prostate cancer: novel scoring systems

**DOI:** 10.1186/s12894-022-01013-8

**Published:** 2022-04-19

**Authors:** Tong Chen, Fei Wang, Hanbing Chen, Meng Wang, Peiqing Liu, Songtao Liu, Yibin Zhou, Qi Ma

**Affiliations:** 1 Departments of Ultrasound, The Second Affiliated Hospital of Soochow University, Suzhou, Jiangsu China; 2 Departments of Urology, The Second Affiliated Hospital of Soochow University, Suzhou, Jiangsu China

**Keywords:** Transrectal ultrasound, Scoring system, Logistic regression model, Prostate cancer, PI-RADS, Decision curve analysis

## Abstract

**Background:**

To evaluate the diagnostic performance of multiparametric transrectal ultrasound (TRUS) and to design diagnostic scoring systems based on four modes of TRUS to predict peripheral zone prostate cancer (PCa) and clinically significant prostate cancer (csPCa).

**Methods:**

A development cohort involved 124 nodules from 116 patients, and a validation cohort involved 72 nodules from 67 patients. Predictors for PCa and csPCa were extracted to construct PCa and csPCa models based on regression analysis of the development cohort. An external validation was performed to assess the performance of models using area under the curve (AUC). Then, PCa and csPCa diagnostic scoring systems were established to predict PCa and csPCa. The diagnostic accuracy was compared between PCa and csPCa scores and PI-RADS V2, using receiver operating characteristics (ROC) and decision curve analysis (DCA).

**Results:**

Regression models were established as follows: PCa = − 8.284 + 4.674 × Margin + 1.707 × Adler grade + 3.072 × Enhancement patterns + 2.544 × SR; csPCa = − 7.201 + 2.680 × Margin + 2.583 × Enhancement patterns + 2.194 × SR. The PCa score ranged from 0 to 6 points, and the csPCa score ranged from 0 to 3 points. A PCa score of 5 or higher and a csPCa score of 3 had the greatest diagnostic performance. In the validation cohort, the AUC for the PCa score and PI-RADS V2 in diagnosing PCa were 0.879 (95% confidence interval [CI] 0.790–0.967) and 0.873 (95%CI 0.778–0.969). For the diagnosis of csPCa, the AUC for the csPCa score and PI-RADS V2 were 0.806 (95%CI 0.700–0.912) and 0.829 (95%CI 0.727–0.931).

**Conclusions:**

The multiparametric TRUS diagnostic scoring systems permitted better identifications of peripheral zone PCa and csPCa, and their performances were comparable to that of PI-RADS V2.

**Supplementary Information:**

The online version contains supplementary material available at 10.1186/s12894-022-01013-8.

## Background

Prostate cancer (PCa) is the most frequently diagnosed cancer among men in most countries of the world [1]. In particular, the incidence and mortality of PCa have recently risen rapidly in China [2]. Early and accurate diagnosis of PCa and the identification of clinically significant PCa (csPCa) can lead to reductions in mortality. However, such early identification remains challenging in clinical practice [3]. Currently, a definitive diagnosis of PCa relies on transrectal ultrasound (TRUS)-guided biopsy [4]. Unfortunately, there is a substantial risk of biopsy-related complications, and a considerable fraction of malignancies are identified only by repeat biopsy [5]. Therefore, developing non-invasive imaging examination techniques to assess prostate lesions is important in order to help avoid unnecessary biopsy.

Multiparametric magnetic resonance imaging (mpMRI) has proven to be particularly valuable for the evaluation of PCa, including detection, staging, and evaluation of aggressiveness [6]. mpMRI is more time-consuming and expensive than TRUS-guided biopsy. A small but significant number of patients are not candidates for MRI because of metal prostheses or claustrophobia. Grayscale US imaging is another technology that has been studied extensively in the realm of PCa diagnosis. However, the sensitivity and specificity of Grayscale US are limited, ranging between 40 and 50% for PCa detection, with minimal additional improvement using Color/Power Doppler [7–9]. Recently, some new US imaging techniques have been developed. In particular, elastography and contrast-enhanced ultrasound (CEUS) may improve both lesion characterization and PCa detection.

Advanced prostate US imaging techniques have undergone incremental improvements in detection of PCa and csPCa, but none currently provides sufficient accuracy when considered separately. Some reports have suggested that multiparametric TRUS may offer an effective alternative for early diagnosis of PCa and csPCa [10, 11]. To our knowledge, however, no report exists regarding the multiparametric use of and diagnostic scoring systems employing all TRUS modes, including Grayscale US, Color Doppler ultrasound (CDUS), CEUS and strain elastography (SE). The aim of this study, then, was to established multiparametric TRUS diagnostic scoring systems and to evaluate the performance of these systems in the diagnosis of peripheral zone PCa and csPCa.

## Methods

### Patients

From January 2016 to March 2021, 116 patients with suspected PCa who were treated at the Second Affiliated Hospital of Soochow University were enrolled as a development cohort in this study. Then, we continuously enrolled 67 patients from the same center as a validation cohort. The inclusion criteria were: (1) patients who received standardized prostate multiparametric TRUS and mpMRI before biopsy; (2) an interval between prostate biopsy and imaging examinations of less than 1 week; (3) performing of standardized prostatic biopsy after examination and the availability of pathological results; (4) availability of PI-RADS V2 results; (5) location of the nodule in the peripheral zone; and (6) lack of radiation therapy, hormonal therapy or other treatments prior to examination. The exclusion criterion were: (1) unsatisfactory quality of images; (2) unable to give informed consent.

### Multiparametric transrectal ultrasound assessment

**Fig. 1:**
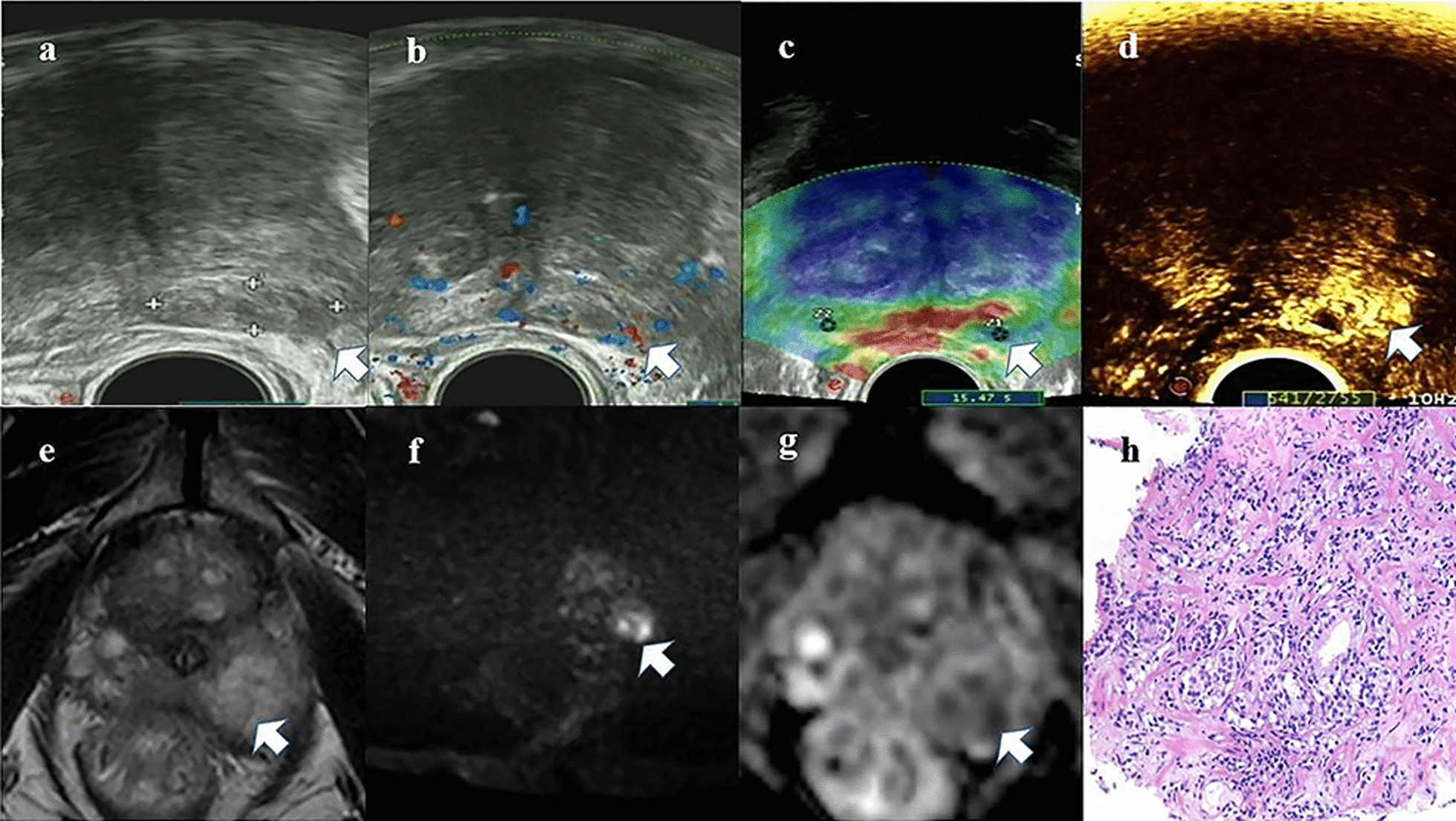
Images from a representative patient (age 61 years). **a** Grayscale US showed a hypoechoic lesion (arrow) in the left peripheral zone with an unclear margin and uneven distribution. **b** The lesion (arrow) was scored as Adler II–III via CDUS. **c** SE imaging was blue in the area of the lesion (arrow). **d** CEUS showed early wash-in/out and high enhancement (arrow). **e** A focal and slightly hypointense lesion was identified in the left posterior peripheral zone with T2-weighted MRI. **f** DWI showed a hyperintense lesion. **g** ADC showed a hypointense lesion. The PI-RADS V2 of this lesion is 5. **h** Histopathological images (× 200). Upon biopsy, the lesion was diagnosed as PCa with a Gleason score of 5 + 4

Multiparametric TRUS was performed for patients presenting with an elevated prostate-specific antigen (PSA) level (> 4 ng/mL). Grayscale US, CDUS, CEUS and SE were performed with a US system (MyLab Twice) using an EC123 transrectal end-fire probe (3–9 MHz) (for examples of transrectal ultrasonic findings, see Fig. 1). The patient was in the left decubitus position. Grayscale US was performed by measuring 3 diameters of the prostate to observe suspicious lesions. The volume of the prostate was calculated by the ellipsoid formula: width × height × length × π/6 (Fig. 1a). Adler grade classifications were conducted based on the blood flow as detected by CDUS [12] (Fig. 1b). Adler grades 0 and I were defined as CDUS negative, while Adler grades II and III were defined as CDUS positive.

The radiologist collected strain elastography images using repeated probe movements and by applying various compression ratios until stable and reproducible images were obtained (Fig. 1c). The strain ratio (SR) was defined as the ratio of Z2 of the surrounding reference tissue that exhibited moderate elasticity (green area) to Z1 of the suspicious lesion (area with repetitive dark blue) (Z2/Z1). The optimal cut-off value was defined as the point with the highest sum of sensitivity and specificity. The cut-off value of SR was 1.69. Then, SR was dichotomized according to the optimal cut-off value.

Finally, CEUS was performed. During CEUS examinations, a fast bolus injection of 2.4 mL of the contrast agent SonoVue (Bracco, Milan, Italy) was administered intravenously. This was followed by administration of 5 mL of a normal saline flush. A video file was recorded to monitor perfusion behavior in the target lesion with time. We examined perfusion patterns of the target lesion compared to those of adjacent tissue. (Fig. 1d). Target lesions with synchronous wash-in or wash-out times and equal enhancements were defined as CEUS negative.

All cases were scanned thoroughly by two radiologists with 10 years of experience in TRUS. A total of 8 TRUS parameters for each lesion was obtained. Values of continuous variables (such as nodule size and SR) were recorded as numbers, and positive or negative results of binary variables (such as echogenicity, distribution, margin, demarcation of internal and external glands, Adler grade and enhancement patterns) were recorded as 1 or 0.

### Multiparametric MRI assessment

All scans were performed using a 3.0 T MR scanner (Philips Intera Achieva, Best, Netherlands) with a 32-channel body phased array coil as the receiving coil. Scan sequences included sagittal T2-weighted images (T2WI), axial T2WI, T1WI, diffusion-weighted images (DWI) (*b* values of 0 and 1000 s/mm^2^) and dynamic contrast-enhanced (DCE) images. Apparent diffusion coefficient maps were calculated on a designated workstation (Fig. 1e–g).

Two radiologists with greater than 10 years of experience in MRI analyses independently interpreted MRI images according to PI-RADS V2 and recorded scores. PI-RADS V2 categories from the 2016 European Society of Urogenital Radiology prostate MRI guidelines [13] were used as diagnostic criteria. A lesion with an overall score of 4 or higher was diagnosed as malignant. During the study period, all reports are based on PI-RADS V2, no version changes are involved.

### Assessment of interrater reliability

The kappa statistic was used to test interrater reliability. Initially, multiparametric TRUS and multiparametric MRI imaging data of 50 patients were randomly selected from the study group. All multiparametric TRUS images and PI-RADS V2 were read independently by two experienced radiologists.

### Prostatic biopsy and pathology assessment

Nodules were first identified by multiparametric TRUS and then they were evaluated by mpMRI. The evaluators of mpMRI images were blind to the multiparametric TRUS results. After the completion of mpMRI analyses, TRUS-guided biopsy was performed with an 18-gauge biopsy gun. Patients with suspected nodules were subjected to both target and systematic prostatic biopsies. Two cores were taken in each index lesion. The pathologist was blinded to the pathology imaging results. The Gleason score was determined for each specimen. A sample image used in histopathology analysis is shown in Fig. 1h.

### Standard of references

The histological grading method for PCa was performed using the Gleason grading system [14]. In particular, csPCa is defined as a tumor with a Gleason score of at least 7 (with either scores of 3 + 4 or 4 + 3) or a volume of at least 0.5 cm^3^. This system is consistent with PI-RADS V2 guidelines [13].

### Development of the model and multiparametric TRUS diagnostic score

Eight TRUS parameters in the PCa and non-PCa groups were compared, and the parameters that were significantly different between the two groups (*P* < 0.05) were selected for the drawing of ROC curves. Parameters with AUC values greater than 0.5 were included in a binary logistic regression approach to establish two models: a PCa model and a csPCa model. Only TRUS parameters were included in the models, and the enter method was used while running the multivariable logistic regression. For each subpopulation, we performed a second logistic regression analysis using the dichotomized explanatory parameters, and then the coefficient of each of these parameters was divided by the smallest coefficient in the model and allocated a weight based on rounding this to the nearest integer. Specifically, the weight for each parameter was defined as the absolute value of the coefficient, and the overall score for each lesion was obtained by summing the weights. The point with highest sum of sensitivity and specificity was used as the diagnostic endpoint.

### Model validation

The PCa and csPCa models were externally validated using the validation cohort regarding predictive accuracy with ROC curves. The AUC was calculated for the PCa and csPCa models using the validation cohort.

### Statistical analysis

Data were analyzed with SPSS version 23.0 software (IBM). Baseline patient data, including age, prostate volume, PSA and prostate-specific antigen density (PSAD) are presented as mean ± standard deviation. Binary parameters were evaluated through *χ*^2^ or Fisher’s exact tests. Continuous variables with normal distributions and equal variances were compared with Student’s t-tests, and variables with unequal variances were compared with Mann–Whitney U-tests. Differences for which *P* < 0.05 were considered statistically significant, and all were bilateral tests. ROC analysis was performed and the AUC was calculated. A Delong test was used to assess differences between AUC results. DCA were performed with Stata 17. PCa and csPCa probability threshold are ranges between the minimum points of decision curve that were just separated from the All line and the maximal points of decision curve that first contacted the None line.

## Results

### Clinical characteristics of patients

**Fig. 2:**
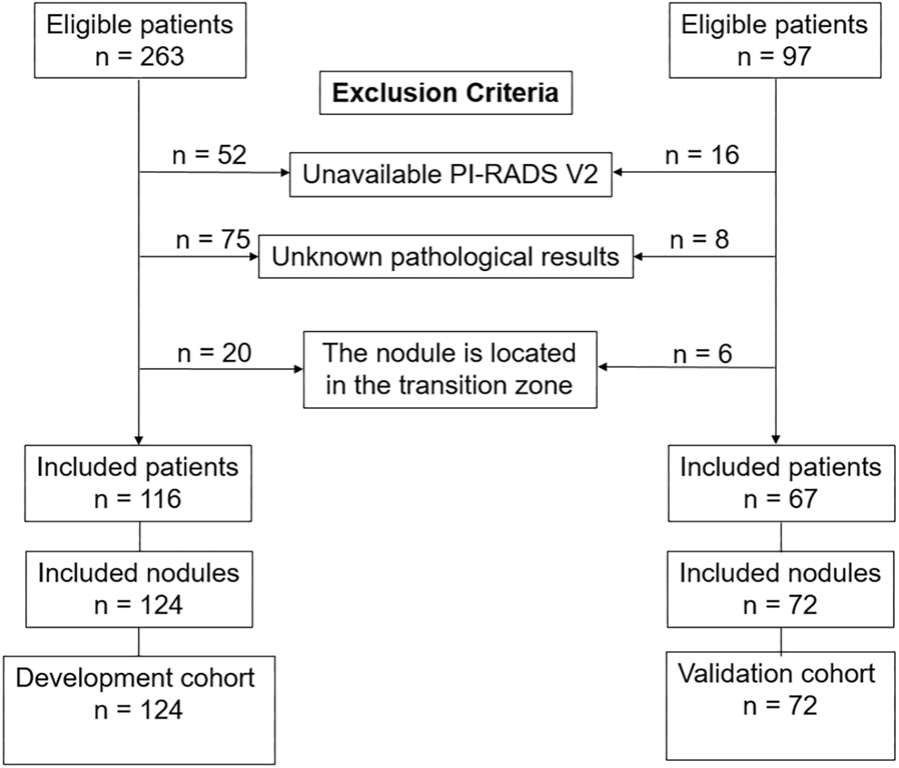
Flow diagram for inclusion of patients into the study

The characteristics of the development and validation cohorts are listed in Table 1. Additional table files show this in more detail [see Additional file 1 and Additional file 2]. A total of 116 patients with a total of 124 prostate nodules were enrolled as a development cohort in this study. A total of 67 patients with a total of 72 prostate nodules were enrolled as a validation cohort. A flowchart of patient recruitment is shown in Fig. 2. Histopathological analyses revealed PCa in 71 (57.3%) of these nodules and non-PCa in 53 (42.7%) nodules in the development cohort. Among the 71 nodules with PCa in the development cohort, 55 were defined as csPCa. In the validation cohort, PCa was identified in 43 (59.7%) of the nodules, and 29 (40.3%) of the nodules were determined to be non-PCa. Among the 43 nodules with PCa in the validation cohort, 37 were defined as csPCa. In the development cohort, MRI defined 74 nodules as PCa. Among them, 5 cases were missed and 8 cases were misdiagnosed. In the validation cohort, MRI defined 47 nodules as PCa, 2 cases were missed and 6 cases were misdiagnosed.

**Table 1:** Characteristics of patients in the development and validation cohorts

Characteristics	Development cohort	Validation cohort
Patients (n)	116	67
Lesions (n)	124	72
Age (years)	71.27 ± 8.50	71.73 ± 8.53
Prostate volume (mL)	61.56 ± 35.14	59.26 ± 27.42
PSA (ng/mL)	19.37 ± 20.45	22.12 ± 29.70
PSAD (ng/mL/cm^3^)	0.38 ± 0.44	0.43 ± 0.59
PCa, n (%)	71 (57.3)	43 (59.7)
csPCa, n (%)	55 (44.4)	37 (51.4)
MRI T-stage (n)		
T2/T3/T4	63/8/NA	34/8/1
MRI N-stage (n)		
N0/N1	66/5	38/5
MRI M-stage (n)		
M0/M1	69/2	39/4
PI-RADS V2 (n)		
2/3/4/5	27/23/39/35	15/10/27/20
Gleason score, n (%)		
3 + 3 = 6	16 (12.9)	6 (8.3)
3 + 4 = 7	12 (9.7)	11 (15.3)
4 + 3 = 7	16 (12.9)	12 (16.7)
4 + 4 = 8	18 (14.5)	5 (6.9)
4 + 5 = 9	3 (2.4)	3 (4.2)
5 + 4 = 9	2 (1.6)	4 (5.6)
5 + 5 = 10	4 (3.2)	2 (2.8)

*PCa* prostate cancer, *csPCa* clinically significant prostate cancer, *PSA* prostate-specific antigen, *PSAD* prostate-specific antigen density, *NA* not available

### Interrater reliability

For the Grayscale US (margin, κ = 0.71, CI 0.50–0.91), Grayscale US (distribution, κ = 0.72, CI 0.53–0.91), CDUS (κ = 0.76, CI 0.62–0.90) and SE (κ = 0.80, CI 0.63–0.97) analyses, the interrater reliabilities were substantial. For the CEUS (κ = 0.84, CI 0.69–0.99), the interrater reliability was almost perfect. For the PI-RADS V2, the interrater reliability was substantial (κ = 0.78, 0.64–0.93). Taken together, these results demonstrate the consistently high agreement among raters and thus support the quality of the present analysis.

### Relationship between TRUS parameters and PCa

Regarding the investigation of PCa by multiparametric TRUS, 8 TRUS parameters were extracted for analysis (Table 2). Six of the eight TRUS parameters were significantly different between the PCa and the non-PCa groups (*P* < 0.05). ROC curves of the 6 significantly different parameters were drawn, and these curves were also found to have AUC values larger than 0.5 (Table 4). The other two parameters (echogenicity and nodule size) were not significantly different between the two groups (*P* > 0.05), which indicates that they are not of diagnostic value for PCa.

**Table 2:** Data from Grayscale US, CDUS, CEUS and SE analyses of PCa and non-PCa nodules

Characteristic	Non-PCa	PCa	*P*
*Grayscale US*			
Echogenicity, n (%)			0.138
Hypoechoic	46 (40.4)	68 (59.6)	
Other echo	7 (70.0)	3 (30.0)	
Margin, n (%)			< 0.001
Clear	22 (95.7)	1 (4.3)	
Unclear	31 (30.7)	70 (69.3)	
Distribution, n (%)			< 0.001
Even	32 (65.3)	17 (34.7)	
Uneven	21 (28.0)	54 (72.0)	
Demarcation of internal and external glands, n (%)			0.031
Clear	46 (47.9)	50 (52.1)	
Unclear	7 (25.0)	21 (75.0)	
Nodule size (mm)	13.00 ± 7.26	15.52 ± 8.81	0.084
*CDUS*			
Adler grade, n (%)			< 0.001
Adler II–III	16 (22.2)	56 (77.8)	
Adler 0–I	37 (71.2)	15 (28.8)	
*CEUS*			
Enhancement patterns, n (%)			< 0.001
Synchronous wash-in or out, equal enhancement	39 (84.8)	7 (15.2)	
Other patterns	14 (17.9)	64 (82.1)	
*SE*			
SR	38.83	80.17	< 0.001

Other patterns included early wash-in or wash-out and high or low enhancement; late wash-in or wash-out and high or low enhancement

*TRUS* transrectal ultrasound, *US* ultrasound, *CEUS* contrast-enhanced ultrasound, *PCa* prostate cancer

### Relationship between TRUS parameters and csPCa

To explore to the diagnostic use of multiparametric TRUS in csPCa, 8 TRUS parameters were extracted for analysis (Table 3). Seven of the eight TRUS parameters were significantly different between the csPCa and non-csPCa groups (*P* < 0.05). ROC curves of the 7 significantly different parameters were drawn and were found to have AUC values larger than 0.5 (Table 4). Echogenicity was not significantly different between the two groups (*P* > 0.05), which suggests that it does not have diagnostic value for csPCa.

**Table 3:** Data from Grayscale US, CDUS, CEUS and SE analyses of csPCa and non-csPCa nodules

Characteristic	Non-csPCa	csPCa	*P*
*Grayscale US*			
Echogenicity, n (%)			0.341
Hypoechoic	62 (54.4)	52 (45.6)	
Other echo	7 (70.0)	3 (30.0)	
Margin, n (%)			< 0.001
Clear	22 (95.7)	1 (4.3)	
Unclear	47 (46.5)	54 (53.5)	
Distribution, n (%)			< 0.001
Even	38 (77.6)	11 (22.4)	
Uneven	31 (41.3)	44 (58.7)	
Demarcation of internal and external glands, n (%)			0.031
Clear	58 (60.4)	38 (39.6)	
Unclear	11 (39.3)	17 (60.7)	
Nodule size (mm)	12.99 ± 7.10	16.27 ± 9.24	0.027
*CDUS*			
Adler grade, n (%)			< 0.001
Adler II–III	26 (36.1)	46 (63.9)	
Adler 0–I	43 (82.7)	9 (17.3)	
*CEUS*			
Enhancement patterns, n (%)			< 0.001
Synchronous wash-in or out, equal enhancement	43 (93.5)	3 (6.5)	
Other patterns	26 (33.3)	52 (66.7)	
*SE*			
SR	45.51	83.82	< 0.001

Other patterns included early wash-in or wash-out and high or low enhancement; late wash-in or wash-out and high or low enhancement

*TRUS* transrectal ultrasound, *US* ultrasound, *CEUS* contrast-enhanced ultrasound, *PCa* prostate cancer, *csPCa* clinically significant prostate cancer

**Table 4:** Area under ROC curve for 7 ultrasound parameters

Parameters	Margin	Distribution	Demarcation of internal and external glands	Adler grade	Enhancement patterns	SR	Nodule size
AUC (PCa)	0.701	0.682	0.582	0.743	0.819	0.823	NA
AUC (csPCa)	0.650	0.675	0.575	0.730	0.784	0.797	0.619

*SR* strain ratio, *PCa* prostate cancer, *csPCa* clinically significant prostate cancer; *NA* not available

### Model development and performance analysis

The regression model of PCa and csPCa was developed based on the 6 and 7 significantly different parameters. The parameters ultimately the finally selected parameters were margin, Alder grade, enhancement patterns and SR for the PCa model, and margin, enhancement patterns and SR for the csPCa model. The resulting models were as follows: PCa = − 8.284 + 4.674 × Margin + 1.707 × Adler grade + 3.072 × Enhancement patterns + 2.544 × SR; csPCa = − 7.201 + 2.680 × Margin + 2.583 × Enhancement patterns + 2.194 × SR. No collinearity among parameters was noted; all tolerances were more than 0.1 and all variance inflation factors were less than 10. The PCa model was found to have an AUC of 0.971 (sensitivity 91.55%, specificity 94.34%, CI 0.924–0.993). The csPCa model was found to have an AUC of 0.921 (sensitivity 87.27%, specificity 89.86%, CI 0.859–0.962). Clearly greater AUCs were obtained for the PCa and csPCa models than for the use of any single parameter alone. The PCa and csPCa models both showed good calibration as assessed by the Hosmer–Lemeshow goodness-of-fit test (*P* = 0.46 and *P* = 0.37, respectively).

### Model validation

In the external validation cohort, the AUC of the PCa model was 0.940 (sensitivity 93.02%, specificity 89.66%, NPV 81.3%, CI 0.858–0.982). The AUC of the csPCa model was 0.880 (sensitivity 89.19%, specificity 80.00%, NPV 78.4%, CI 0.781–0.944).

### Development of multiparametric TRUS diagnostic score

**Fig. 3:**
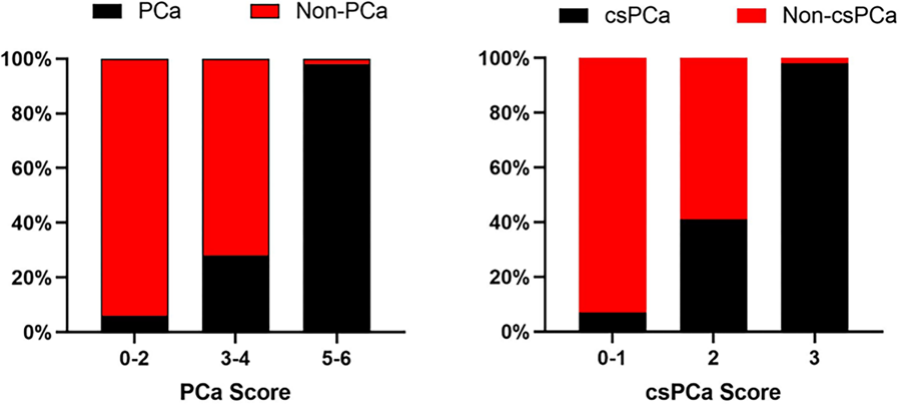
Bar plots showing the increasing detection of PCa and csPCa according to the points in each score

For the PCa score, we weighted the parameters by attributing 1 point to Adler grade and SR, and 2 points to margin and enhancement patterns, with a final score ranging from 0 to 6 points (Table 5). For the csPCa score, we weighted the parameters by attributing 1 point to margin, enhancement patterns and SR, with a final score ranging from 0 to 3 points (Table 5). Enhancement patterns has higher association to predict PCa (*P* < 0.001) and csPCa (*P* = 0.001). The diagnostic accuracy of different cutoff values of the PCa score and the csPCa score are shown in Table 6. A lesion with an overall PCa score of 5 or higher was diagnosed as PCa, whereas a lesion with an overall csPCa score of 3 or higher was diagnosed as csPCa. Bar plots showed the stepwise increase in risk of PCa and csPCa for increasing values of scores (Fig. 3). The AUC for the PCa score in diagnosing PCa was 0.920 (CI 0.867–0.973). For the diagnosis of csPCa, the AUC for the csPCa score was 0.871 (CI 0.802–0.940).

**Table 5:** Binary logistic regression of multiparametric TRUS parameters and details about calculation of the PCa and csPCa scores

	B	Odds Ratio	CI 95%	*P*	Points
*PCa score*					
Margin	4.674	107.11	6.36–1798.00	0.001	2
Alder grade	1.707	5.51	1.17–26.06	0.031	1
Enhancement patterns	3.072	21.59	4.68–99.82	< 0.001	2
SR	2.544	12.73	2.75–59.02	0.001	1
*csPCa score*					
Margin	2.680	12.70	1.14–141.55	0.042	1
Enhancement patterns	2.583	13.77	3.25–58.31	0.001	1
SR	2.194	8.89	2.20–35.98	0.002	1

*CI* confidence interval, *SR* strain ratio

**Table 6:** Diagnostic accuracy of different Cut-Offs of PCa score and csPCa score

	Sensitivity, %	Specificity, %	FP	FN	TP	TN
*PCa score*						
≥ 0	100.0 (93.6–100.0)	0.0 (0.0–6.7)	53 (42.7)	0 (0.0)	71 (57.3)	0 (0.0)
≥ 1	100.0 (93.6–100.0)	15.1 (7.1–28.1)	45 (36.3)	0 (0.0)	71 (57.3)	8 (6.4)
≥ 2	100.0 (93.6–100.0)	26.4 (15.7–40.6)	39 (31.5)	0 (0.0)	71 (57.3)	14 (11.3)
≥ 3	97.2 (89.3–99.5)	58.5 (44.2–71.6)	22 (17.7)	2 (1.6)	69 (55.6)	31 (25.0)
≥ 4	95.8 (87.3–98.9)	79.2 (65.5–88.7)	11 (8.9)	3 (2.4)	68 (54.8)	42 (33.9)
≥ 5	85.9 (75.2–92.7)	98.1 (88.6–99.9)	1 (0.8)	10 (8.1)	61 (49.2)	52 (41.9)
= 6	66.2 (53.9–76.7)	100.0 (91.6–100.0)	0 (0.0)	24 (19.4)	47 (37.9)	53 (42.7)
*csPCa score*						
≥ 0	100.0 (91.9–100.0)	0.0 (0.0–6.6)	69 (55.6)	0 (0.0)	55 (44.4)	0 (0.0)
≥ 1	100.0 (91.9–100.0)	18.8 (10.8–30.4)	56 (45.2)	0 (0.0)	55 (44.4)	13 (10.5)
≥ 2	98.2 (89.0–99.9)	56.5 (44.1–68.2)	30 (24.2)	1 (0.8)	54 (43.5)	39 (31.5)
= 3	87.3 (74.9–94.3)	87.0 (76.2–93.5)	9 (7.3)	7 (5.6)	48 (38.7)	60 (48.4)

*FN* false negative, *FP* false positive, *TN* true negative, *TP* true positive

### Comparison of the diagnostic performances of the model with that of PI-RADS V2

**Fig. 4:**
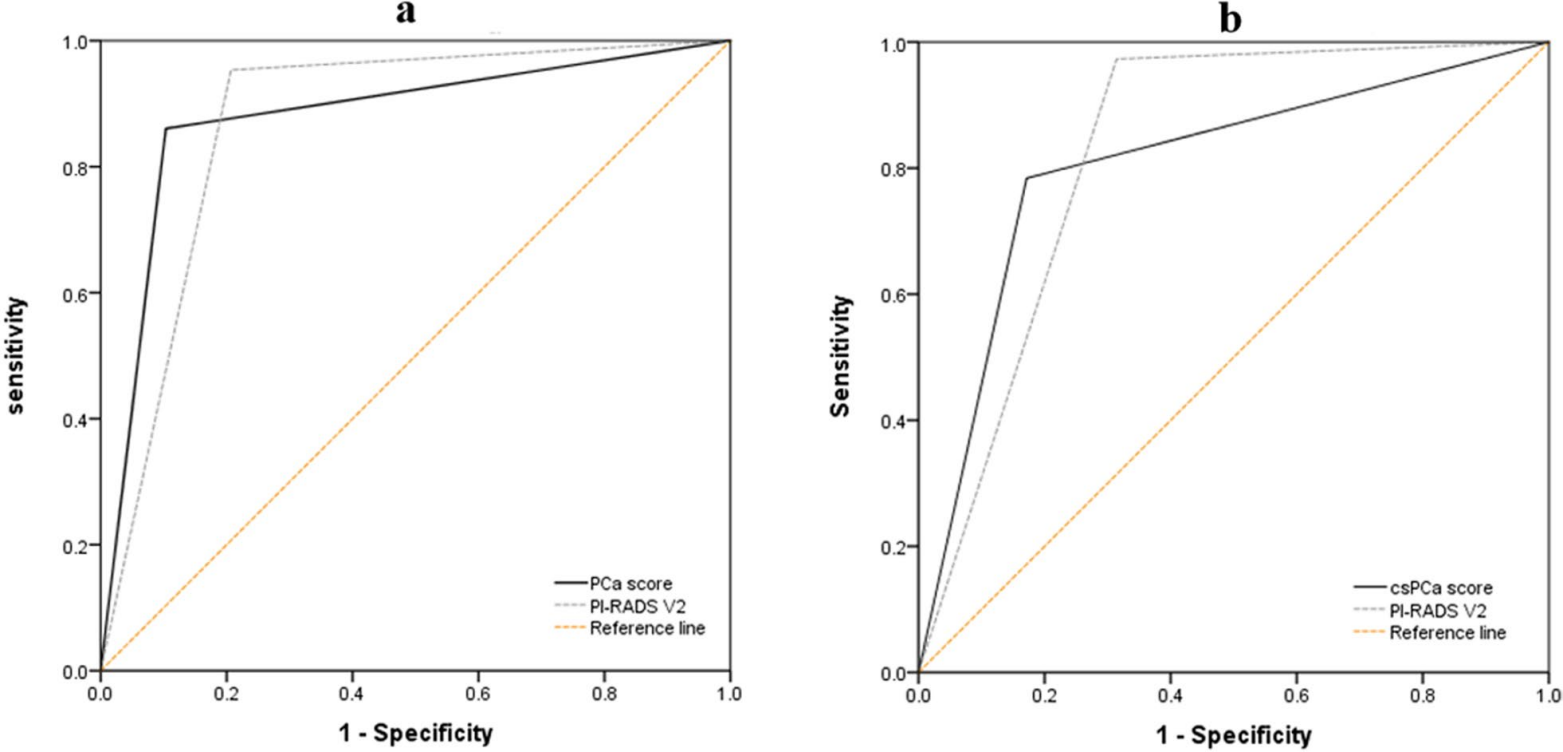
Comparison of ROC curves between multiparametric TRUS scoring systems and PI-RADS V2 score for the detection of **a** prostate cancer and **b** clinically significant prostate cancer

In the external validation cohort, the AUC for the PCa score and for PI-RADS V2 in diagnosing PCa were 0.879 (CI 0.790–0.967) and 0.873 (CI 0.778–0.969), respectively (*P* > 0.05) (Fig. 4a). For the diagnosis of csPCa, the AUC for the csPCa score and for PI-RADS V2 were 0.806 (CI 0.700–0.912) and 0.829 (CI 0.727–0.931), respectively (*P* > 0.05) (Fig. 4b). As a result, PCa and csPCa scores were comparable with PI-RADS V2 in diagnosing PCa and csPCa.

### Decision curve analysis

**Fig. 5:**
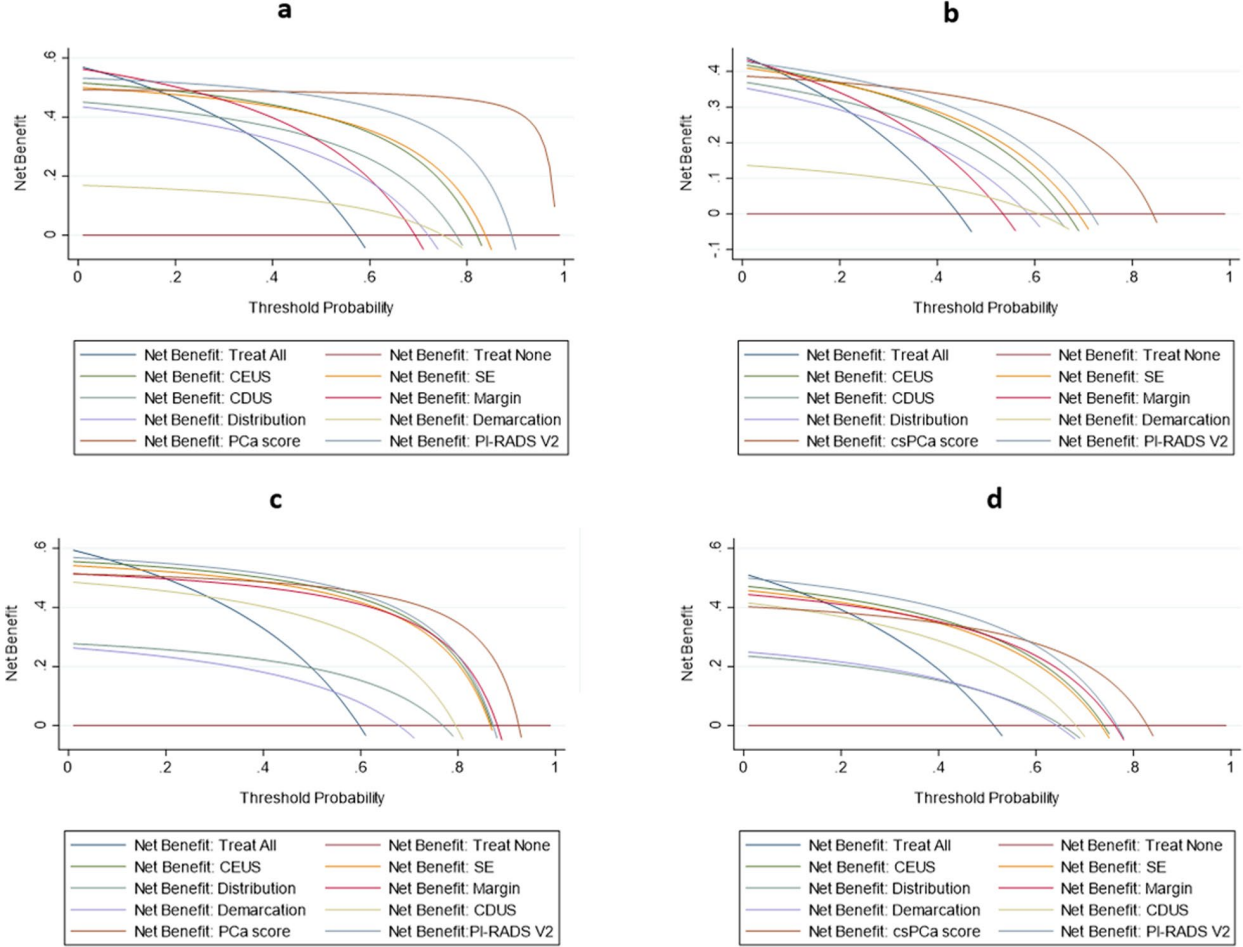
Decision curve analysis. **a**, **b** PCa and csPCa scores in the development cohort. **c**, **d** PCa and csPCa scores in the validation cohort. The blue line (Net Benefit: Treat All) was the hypothesis that all patients had PCa and csPCa and the red line (Net Benefit: Treat None) which parallel to the horizontal axis was the hypothesis that no patients had PCa and csPCa

According to the DCA, in the development cohort, ranges of threshold probabilities from 15 to 100% for PCa and from 10 to 85% for csPCa were predictive of a net benefit of using PCa and csPCa scores for the diagnosis of PCa and csPCa. For PCa and csPCa, when threshold probabilities ranged from 42 to 100% and 30–85%, respectively, the related PCa and csPCa scores became more benefit as compared with PI-RADS V2 (Fig. 5a, b). In the validation cohort, when threshold probabilities ranged from 20 to 92% for PCa and 22–83% for csPCa, a net positive benefit was found for diagnosis. In addition, when threshold probabilities ranged from 60 to 92% for PCa and 59–83% for csPCa, the related PCa and csPCa scores were more benefit as compared with PI-RADS V2 (Fig. 5c, d).

## Discussion

Conventional screening methods for PCa include prostate-specific antigen testing and digital rectal examination. Imaging methods, however, are currently accepted as optimal for assessing prostate cancer, as they are less invasive than conventional methods and can be more sensitive. Advanced prostate US in particular takes advantage of the benefits of improved grayscale US, vascular imaging techniques, US elastography and perfusion imaging [15]. While these combinations have improved diagnostic results, no studies have yet combined together all TRUS techniques, including grayscale US, CDUS, CEUS and SE.

Before our study, five-point subjective scale has been reported by Halpern, et al. [16], in which detailed evaluations for Grayscale US, Doppler flow and CEUS were provided. In this work, Grayscale US and CDUS findings were graded as normal, probably normal, indeterminate, probably carcinoma, or definitely carcinoma, and enhancement patterns were graded as minimal, mild, mildly increased, moderately increased, or substantially increased. However, it ignored the value of each parameter in the diagnosis of PCa. Specifically, parameters with high diagnostic value should be assigned a higher weight. Here, then, we focused on the assessment of multiparametric TRUS scoring systems in the diagnosis of PCa and csPCa.

In this study, in total, 8 TRUS parameters out of the four modes (Grayscale US, CDUS, CEUS and SE) were obtained. Ultimately, a regression model based on 6 significant parameters in PCa and a regression model based on 7 significant parameters in csPCa were established in the development cohort. The diagnostic performance of both models was then validated using a validation cohort. In addition, we designed 2 simple multiparametric scores, including the PCa score and the csPCa score, to diagnose these two diseases.

In patients with PCa, the parameters individually associated with the highest diagnostic accuracy were margin, Adler grade, enhancement patterns and SR. The good diagnostic accuracy of parameters involving CDUS, CEUS, and SR is not surprising. CDUS is well known to be able to reveal an increased number of visualized vessels as well as an increase in flow rate, size, and irregularity of vessels within a PCa lesion [17]. CEUS allows a visualization of the microvascularization, which leads CEUS to be widely used to enhance the visualization of perfusion changes related to PCa [18]. Studies by Halpern et al. [19] and Maxeiner et al. [20] have shown that CEUS is more applicable for quantitative detection of hemodynamics as compared with CDUS. Accordingly, the coefficient of CEUS was twice that of CDUS in the PCa model that we developed.

PCa tissue is stiffer than surrounding normal tissue due to several changes, including increased cellular density, destruction of glandular architecture, and development of stromal reactions combined with collagen deposition in the surrounding prostate parenchyma [21]. Thus, SE has the potential to clearly distinguish features of lesions and thus to be of great value for the detection of PCa. Specifically, the measurement of SR can be diagnostic, as PCa tissues often show either partial compression or no obvious compression, leading to increased SR.

In patients with csPCa, the parameters associated with the best diagnostic accuracy were margin, enhancement patterns and SR. Here, we focused more on the exclusion of CDUS from the csPCa model. CDUS reflects increased tumor blood vessels, but as lymph nodes increase as the tumor progresses, the ratio of the tumor volume to the total blood volume decreases, which may lead to less accurate CDUS scores.

These analyses confirm that the various TRUS parameters are associated with a variable degree of diagnostic accuracy and that a combination of parameters has the potential to increase the diagnostic accuracy of PCa and csPCa. The research of Mingbo et al. [10] also demonstrated that multiparametric TRUS has high diagnostic performance in the diagnosis of localized PCa. Another study showed that multiparametric US significantly improved the detection of PCa lesions with Gleason scores of 3 + 4 = 7 or greater compared to a single US parameter [11].

PCa score showed very good diagnostic accuracy in identifying patients with PCa, with an AUC of 0.920. Because confirming or excluding PCa has a significant impact on biopsy and treatment strategies, we propose the use of highly sensitive and highly specific cutoff scores (PCa score < 1 and ≥ 5) to exclude or confirm the diagnosis of PCa. csPCa score also showed very good diagnostic accuracy in identifying patients with csPCa, with an AUC of 0.806. We propose the use of a highly sensitive and highly specific cutoff score (csPCa score = 3) to confirm the diagnosis of csPCa. Mannaerts et al. believe that multiparametric US had significantly improved sensitivity for all csPCa definitions at the 2 Likert threshold, with a sensitivity of 74–85%, when considering a Likert score of 3 or greater to be suspicious [11]. Our study results are similar to this result, in that the sensitivity of our csPCa score definitions was 87.3%.

We futher investigated PCa and csPCa scores for identifying PCa and csPCa with an AUC of 0.879 and 0.806 in the validation cohort. Moreover, we analyzed the diagnostic performance of the PCa and csPCa scores as compared with PI-RADS V2. PCa score performance may be similar to that of PI-RADS V2 0.879 (95% CI 0.790–0.967) vs. 0.873 (95% CI 0.778–0.969) (*P* > 0.05). These results are consistent with those of the study of Mingbo et al. [10] (mpUS: 0.874 ± 0.043, 95% CI 0.790–0.959 vs. mpMRI: 0.774 ± 0.055, 95% CI 0.666–0.881). The diagnostic performance of the csPCa score was also comparable with that of PI-RADS V2. For both the PCa and csPCa scores, higher values were associated with higher PCa and csPCa burdens.

Based on results of DCA, patients with a 20–92% probability threshold in the PCa and a 22–83% probability threshold in the csPCa should be encouraged to undergo prostatic biopsy for further evaluation. Similarly, for patients with a 60–92% probability threshold in the PCa and a 59–83% probability threshold in the csPCa, clinicians should have more confidence in identifying PCa and csPCa using the multiparametic TRUS scoring system.

Multiparametric TRUS currently lacks the standardization and consistency that PI-RADS V2 provides for mpMRI. As an initial exploration, our study established multiparametric TRUS diagnostic scoring systems, which may be seen as an important first step for multicenter standardization, evaluation and implementation of multiparametric TRUS in the clinical setting.

However, our scoring systems still need to be improved. For example, limitations exists have been identified regarding systematic evaluations using that use real-time elastography identified to identify prostate cancer [22]. In particular, sensitivity was found to be lower in ventral areas (30.2–35%) as compared to dorsal areas (31.5–88.9%). Therefore, adding of the prostate sector to scoring systems when assigning weights may make these tools more suitable. For example, elastography results from lesions located in ventral areas should assigned a lower weight.

In addition, quantitative analysis of the CEUS was used in the CADMUS trial [23]. This may provide more valuable parameters for scoring systems.

There were several limitations to this study. First, the current present study has used a relatively small sample size and is a retrospective, single-center study. Therefore, larger sample sizes with populations including diverse demographics from multiple institutions or race are necessary to validate our preliminary primary findings. Second, we did not include transition zone PCa and csPCa. Third, no pathological results of radical prostatectomy specimens were available for reference. Further research will continue to refine these diagnostic tools.

## Conclusions

We proposed regression models and diagnostic scoring systems based on multiparametric TRUS to assess peripheral zone PCa and csPCa and to improve the diagnostic performance of imaging techniques. The diagnostic power of these models is comparable to that of PI-RADS V2 analyses.

## Supplementary Information

**Additional file 1**. Lesions variables in the development cohort, shows all TRUS parameters and preoperative variables for each lesion in the development cohort.

**Additional file 2**. Lesions variables in the validation cohort, shows all TRUS parameters and preoperative variables for each lesion in the validation cohort.

## Data Availability

All data generated or analysed during this study are included in this published article.

## Abbreviations

TRUS: Transrectal ultrasound
PCa: Prostate cancer
csPCa: Clinically significant prostate cancer
PI-RADS V2: Prostate imaging reporting and data system version 2
AUC: Area under the curve
ROC: Receiver operating characteristic
DCA: Decision curve analysis
CI: Confidence interval
CEUS: Contrast-enhanced ultrasound
CDUS: Color Doppler ultrasound
SE: Strain elastography
mpMRI: Multiparametric magnetic resonance imaging
US: Ultrasound
T2WI: T2-weighted images
DWI: Diffusion-weighted images
DCE: Dynamic contrast-enhanced
PSA: Prostate-specific antigen
PSAD: Prostate-specific antigen density
NA: Not available

## References

[1] Bray F, Ferlay J, Soerjomataram I, Siegel RL, Torre LA, Jemal A (2018). Global cancer statistics 2018: GLOBOCAN estimates of incidence and mortality worldwide for 36 cancers in 185 countries. CA Cancer J Clin.

[2] Zhu Y, Wang HK, Qu YY, Ye DW (2015). Prostate cancer in East Asia: evolving trend over the last decade. Asian J Androl.

[3] Xu L, Zhang G, Shi B, Liu Y, Zou T, Yan W (2019). Comparison of biparametric and multiparametric MRI in the diagnosis of prostate cancer. Cancer Imaging.

[4] Heidenreich A, Bastian PJ, Bellmunt J, Bolla M, Joniau S, van der Kwast T (2014). EAU guidelines on prostate cancer. Part 1: screening, diagnosis, and local treatment with curative intent-update 2013. Eur Urol.

[5] Ukimura O, Coleman JA, de la Taille A, Emberton M, Epstein JI, Freedland SJ (2013). Contemporary role of systematic prostate biopsies: indications, techniques, and implications for patient care. Eur Urol.

[6] Ueno Y, Tamada T, Bist V, Reinhold C, Miyake H, Tanaka U (2016). Multiparametric magnetic resonance imaging: current role in prostate cancer management. Int J Urol.

[7] Norberg M, Egevad L, Holmberg L, Sparén P, Norlén BJ, Busch C (1997). The sextant protocol for ultrasound-guided core biopsies of the prostate underestimates the presence of cancer. Urology.

[8] Beerlage HP, Aarnink RG, Ruijter ET, Witjes JA, Wijkstra H, Van De Kaa CA (2001). Correlation of transrectal ultrasound, computer analysis of transrectal ultrasound and histopathology of radical prostatectomy specimen. Prostate Cancer Prostatic Dis.

[9] Cheng S, Rifkin MD (2001). Color Doppler imaging of the prostate: important adjunct to endorectal ultrasound of the prostate in the diagnosis of prostate cancer. Ultrasound Q.

[10] Zhang M, Tang J, Luo Y, Wang Y, Wu M, Memmott B (2019). Diagnostic performance of multiparametric transrectal ultrasound in localized prostate cancer: a comparative study with magnetic resonance imaging. J Ultrasound Med.

[11] Mannaerts CK, Wildeboer RR, Remmers S, van Kollenburg RAA, Kajtazovic A, Hagemann J (2019). Multiparametric ultrasound for prostate cancer detection and localization: correlation of B-mode, shear wave elastography and contrast enhanced ultrasound with radical prostatectomy specimens. J Urol.

[12] Che D, Yang Z, Wei H, Wang X, Gao J (2020). The Adler grade by Doppler ultrasound is associated with clinical pathology of cervical cancer: Implication for clinical management. PLoS ONE.

[13] Weinreb JC, Barentsz JO, Choyke PL, Cornud F, Haider MA, Macura KJ (2016). PI-RADS prostate imaging—reporting and data system: 2015, version 2. Eur Urol.

[14] Epstein JI, Amin MB, Reuter VE, Humphrey PA (2017). Contemporary gleason grading of prostatic carcinoma: an update with discussion on practical issues to implement the 2014 international society of urological pathology (ISUP) consensus conference on gleason grading of prostatic carcinoma. Am J Surg Pathol.

[15] Palmeri ML, Glass TJ, Miller ZA, Rosenzweig SJ, Buck A, Polascik TJ (2016). Identifying clinically significant prostate cancers using 3-D in vivo acoustic radiation force impulse imaging with whole-mount histology validation. Ultrasound Med Biol.

[16] Halpern EJ, Ramey JR, Strup SE, Frauscher F, McCue P, Gomella LG (2005). Detection of prostate carcinoma with contrast-enhanced sonography using intermittent harmonic imaging. Cancer.

[17] Fleischer AC, Rodgers WH, Rao BK, Kepple DM, Worrell JA, Williams L (1991). Assessment of ovarian tumor vascularity with transvaginal color Doppler sonography. J Ultrasound Med.

[18] Huang H, Zhu ZQ, Zhou ZG, Chen LS, Zhao M, Zhang Y (2016). Contrast-enhanced transrectal ultrasound for prediction of prostate cancer aggressiveness: the role of normal peripheral zone time-intensity curves. Sci Rep.

[19] Halpern EJ, Verkh L, Forsberg F, Gomella LG, Mattrey RF, Goldberg BB (2000). Initial experience with contrast-enhanced sonography of the prostate. AJR Am J Roentgenol.

[20] Maxeiner A, Stephan C, Durmus T, Slowinski T, Cash H, Fischer T (2015). Added value of multiparametric ultrasonography in magnetic resonance imaging and ultrasonography fusion-guided biopsy of the prostate in patients with suspicion for prostate cancer. Urology.

[21] Correas JM, Halpern EJ, Barr RG, Ghai S, Walz J, Bodard S (2021). Advanced ultrasound in the diagnosis of prostate cancer. World J Urol.

[22] Brock M, Eggert T, Palisaar RJ, Roghmann F, Braun K, Löppenberg B (2013). Multiparametric ultrasound of the prostate: adding contrast enhanced ultrasound to real-time elastography to detect histopathologically confirmed cancer. J Urol.

[23] Grey A, Scott R, Charman S, van der Meulen J, Frinking P, Acher P (2018). The CADMUS trial—multi-parametric ultrasound targeted biopsies compared to multi-parametric MRI targeted biopsies in the diagnosis of clinically significant prostate cancer. Contemp Clin Trials.

