# Supplementary material for: Multiparametric transrectal ultrasound for the diagnosis of peripheral zone prostate cancer and clinically significant prostate cancer: novel scoring systems
Source: BMC Urol. 2022 Apr 19;22:64. doi: 10.1186/s12894-022-01013-8 (PMC9016931; doi:10.1186/s12894-022-01013-8)
Supplement: Supplementary file 1 — Additional file 1. Lesions variables in the development cohort, shows all TRUS parameters and preoperative variables for each lesion in the development cohort. [file 12894_2022_1013_MOESM1_ESM.docx]

Additional file 1. Lesions variables in the development cohort.

| Lesions  number | PSA  (ng/mL) | PSAD  (ng/mL/cm^3^) | PV  (mL) | echogenicity | distribution | margin | demarcation of internal and external | Size  (mm) |
| --- | --- | --- | --- | --- | --- | --- | --- | --- |
| 1 | 5.86 | 0.17 | 35.5 | Hypoechoic | Uneven | Unclear | clear | 9 |
| 2 | 9.71 | 0.35 | 28.1 | Hypoechoic | Uneven | Unclear | clear | 12 |
| 3 | 2.34 | 0.05 | 47.5 | Other echo | Uneven | Unclear | clear | 18 |
| 4 | 12.00 | 0.29 | 40.9 | Hypoechoic | Uneven | Unclear | clear | 12 |
| 5 | 5.10 | 0.11 | 47.2 | Hypoechoic | Uneven | Unclear | clear | 7 |
| 6 | 19.92 | 0.47 | 42.4 | Hypoechoic | Uneven | Unclear | clear | 12 |
| 7 | 14.28 | 0.37 | 38.8 | Hypoechoic | Uneven | Unclear | clear | 7 |
| 8 | 1.40 | 0.07 | 20.6 | Hypoechoic | Uneven | Unclear | clear | 8 |
| 9 | 31.62 | 0.44 | 72.2 | Hypoechoic | even | Unclear | clear | 11 |
| 10 | ＞100 | 1.89 | 52.9 | Hypoechoic | Uneven | Unclear | Unclear | 40 |
| 11 | 1.96 | 0.03 | 69.5 | Other echo | Uneven | Unclear | Unclear | 27 |
| 12 | 40.05 | 0.85 | 47.1 | Hypoechoic | Uneven | Unclear | Unclear | 24 |
| 13 | 60.53 | 0.47 | 130.1 | Hypoechoic | even | Unclear | Unclear | 42 |
| 14 | 77.23 | 2.82 | 27.4 | Hypoechoic | Uneven | Unclear | clear | 14 |
| 15 | 5.94 | 0.10 | 31.9 | Hypoechoic | Uneven | Unclear | Unclear | 9 |
| 16 | 10.53 | 0.33 | 31.9 | Hypoechoic | Uneven | Unclear | clear | 15 |
| 17 | - | - | - | Hypoechoic | Uneven | Unclear | clear | 9 |
| 18 | 4.82 | 0.23 | 21.4 | Hypoechoic | even | Unclear | clear | 9 |
| 19 | 4.71 | 0.14 | 33.1 | Hypoechoic | Uneven | Unclear | Unclear | 13 |
| 20 | 8.25 | 0.09 | 96.4 | Hypoechoic | even | Unclear | clear | 11 |
| 21 | 17.44 | 0.71 | 24.4 | Hypoechoic | Uneven | Unclear | clear | 15 |
| 22 | 10.35 | 0.24 | 43.6 | Hypoechoic | Uneven | Unclear | Unclear | 17 |
| 23 | 21.61 | 0.62 | 34.8 | Hypoechoic | Uneven | Unclear | clear | 12 |
| 24 | 38.73 | 0.54 | 71.7 | Hypoechoic | Uneven | Unclear | clear | 23 |
| 25 | 7.99 | 0.18 | 44.0 | Hypoechoic | Uneven | Unclear | clear | 11 |
| 26 | 10.14 | 0.32 | 32.1 | Hypoechoic | Uneven | Unclear | clear | 5 |
| 27 | 2.55 | 0.04 | 56.7 | Hypoechoic | even | Unclear | clear | 13 |
| 28 | 19.31 | 0.28 | 69.1 | Hypoechoic | Uneven | Unclear | Unclear | 27 |
| 29 | 20.96 | 0.44 | 47.8 | Hypoechoic | Uneven | Unclear | clear | 17 |
| 30 | 15.01 | 0.36 | 42.2 | Hypoechoic | Uneven | Unclear | Unclear | 12 |
| 31 | 3.29 | 0.06 | 57.0 | Hypoechoic | even | Unclear | clear | 14 |
| 32 | 58.33 | 1.05 | 55.7 | Hypoechoic | Uneven | Unclear | Unclear | 36 |
| 33 | 41.56 | 0.86 | 48.3 | Hypoechoic | Uneven | Unclear | clear | 19 |
| 34 | 13.00 | 0.36 | 36.3 | Hypoechoic | Uneven | Unclear | clear | 9 |
| 35 | 16.67 | 0.54 | 30.9 | Hypoechoic | Uneven | Unclear | clear | 17 |
| 36 | ＞100 | 1.66 | 56.3 | Hypoechoic | Uneven | Unclear | Unclear | 30 |
| 37 | 58.48 | 1.26 | 46.4 | Other echo | Uneven | Unclear | clear | 13 |
| 38 | 6.38 | 0.12 | 55.1 | Hypoechoic | even | Unclear | clear | 11 |
| 39 | 6.58 | 0.18 | 36.9 | Hypoechoic | even | Unclear | clear | 9 |
| 40 | 1.48 | 0.03 | 44.1 | Hypoechoic | Uneven | Unclear | clear | 26 |
| 41 | 18.25 | 0.11 | 167.0 | Hypoechoic | Uneven | Unclear | clear | 30 |
| 42 | 7.34 | 0.22 | 33.3 | Hypoechoic | Uneven | Unclear | Unclear | 17 |
| 43 | 4.76 | 0.11 | 41.9 | Hypoechoic | even | Unclear | clear | 9 |
| 44 | 36.76 | 0.54 | 68.7 | Hypoechoic | Uneven | Unclear | clear | 28 |
| 45 | 23.38 | 0.41 | 56.8 | Hypoechoic | Uneven | Unclear | Unclear | 28 |
| 46 | 13.06 | 0.29 | 44.6 | Hypoechoic | Uneven | Unclear | clear | 8 |
| 47 | 8.83 | 0.28 | 32.1 | Hypoechoic | Uneven | Unclear | clear | 14 |
| 48 | 12.21 | 0.19 | 62.8 | Hypoechoic | Uneven | Unclear | clear | 8 |
| 49 | 11.43 | 0.20 | 58.0 | Hypoechoic | Uneven | Unclear | clear | 10 |
| 50 | 8.42 | 0.30 | 28.1 | Hypoechoic | Uneven | Unclear | Unclear | 8 |
| 51 | 7.36 | 0.14 | 51.1 | Hypoechoic | Uneven | Unclear | clear | 12 |
| 52 | 13.73 | 0.43 | 31.9 | Hypoechoic | even | Unclear | clear | 10 |
| 53 | 10.53 | 0.25 | 41.5 | Hypoechoic | even | Unclear | clear | 8 |
| 54 | 16.52 | 0.28 | 58.4 | Hypoechoic | even | Unclear | clear | 11 |
| 55 | 35.08 | 0.51 | 68.5 | Hypoechoic | Uneven | Unclear | clear | 10 |
| 56 | 59.47 | 1.32 | 45.0 | Hypoechoic | Uneven | Unclear | clear | 12 |
| 57 | 26.02 | 1.19 | 21.9 | Hypoechoic | Uneven | Unclear | clear | 10 |
| 58 | 34.74 | 1.47 | 23.7 | Hypoechoic | even | Unclear | Unclear | 8 |
| 59 | 51.44 | 1.11 | 46.4 | Hypoechoic | Uneven | Unclear | clear | 12 |
| 60 | - | - | - | Hypoechoic | Uneven | Unclear | Unclear | 12 |
| 61 | - | - | - | Hypoechoic | even | Unclear | Unclear | 7 |
| 62 | - | - | - | Hypoechoic | even | Unclear | clear | 14 |
| 63 | 26.17 | 0.74 | 47.7 | Hypoechoic | Uneven | Unclear | Unclear | 7 |
| 64 | 22.46 | 0.37 | 61.0 | Hypoechoic | Uneven | Unclear | clear | 46 |
| 65 | 18.90 | 0.43 | 43.6 | Hypoechoic | even | Unclear | clear | 18 |
| 66 | 24.33 | 0.59 | 41.1 | Hypoechoic | Uneven | Unclear | Unclear | 20 |
| 67 | 38.29 | 0.80 | 62.9 | Hypoechoic | Uneven | clear | clear | 20 |
| 68 | 22.46 | 0.37 | 61.0 | Hypoechoic | even | Unclear | clear | 16 |
| 69 | 18.90 | 0.43 | 50.2 | Hypoechoic | Uneven | Unclear | Unclear | 20 |
| 70 | 24.33 | 0.59 | 31.9 | Hypoechoic | Uneven | Unclear | Unclear | 8 |
| 71 | 90.57 | 1.44 | 35.3 | Hypoechoic | Uneven | Unclear | clear | 16 |
| 72 | 5.76 | 0.06 | 95.7 | Hypoechoic | Uneven | clear | Unclear | 14 |
| 73 | 19.40 | 0.43 | 45.5 | Hypoechoic | even | clear | Unclear | 15 |
| 74 | 1.53 | 0.04 | 38.1 | Hypoechoic | Uneven | clear | clear | 13 |
| 75 | 15.02 | 0.12 | 130.3 | Hypoechoic | Uneven | clear | clear | 42 |
| 76 | 13.42 | 0.09 | 154.9 | Hypoechoic | Uneven | clear | clear | 7 |
| 77 | 5.51 | 0.06 | 99.1 | Hypoechoic | Uneven | clear | clear | 15 |
| 78 | 10.77 | 0.21 | 52.4 | Hypoechoic | Uneven | Unclear | clear | 18 |
| 79 | 3.84 | 0.04 | 94.1 | Hypoechoic | Uneven | Unclear | clear | 15 |
| 80 | 11.64 | 0.23 | 51.0 | Hypoechoic | even | clear | clear | 15 |
| 81 | 5.55 | 0.14 | 38.6 | Hypoechoic | even | clear | clear | 29 |
| 82 | 62.38 | 1.04 | 59.9 | Hypoechoic | even | clear | Unclear | 31 |
| 83 | 6.80 | 0.07 | 94.8 | Other echo | Uneven | clear | clear | 12 |
| 84 | 17.55 | 0.10 | 173.4 | Hypoechoic | even | Unclear | clear | 7 |
| 85 | 3.96 | 0.06 | 63.6 | Hypoechoic | even | clear | clear | 8 |
| 86 | 15.01 | 0.10 | 148.4 | Hypoechoic | Uneven | Unclear | clear | 15 |
| 87 | 47.79 | 0.80 | 60.0 | Hypoechoic | Uneven | Unclear | clear | 14 |
| 88 | 14.66 | 0.11 | 137.7 | Hypoechoic | Uneven | clear | Unclear | 10 |
| 89 | 7.47 | 0.10 | 73.0 | Hypoechoic | even | clear | clear | 10 |
| 90 | 10.38 | 0.12 | 85.0 | Hypoechoic | even | clear | clear | 7 |
| 91 | 5.20 | 0.12 | 42.3 | Hypoechoic | Uneven | clear | clear | 12 |
| 92 | 7.62 | 0.08 | 101.5 | Hypoechoic | even | Unclear | clear | 13 |
| 93 | 13.20 | 0.43 | 31.0 | Hypoechoic | even | Unclear | clear | 29 |
| 94 | 6.95 | 0.07 | 103.9 | Other echo | even | Unclear | clear | 8 |
| 95 | 15.08 | 0.42 | 36.3 | Hypoechoic | even | clear | clear | 10 |
| 96 | 26.75 | 0.48 | 56.2 | Hypoechoic | even | clear | Unclear | 9 |
| 97 | 5.21 | 0.06 | 85.8 | Other echo | even | clear | clear | 11 |
| 98 | 5.59 | 0.08 | 66.3 | Hypoechoic | Uneven | clear | clear | 6 |
| 99 | 19.87 | 0.18 | 111.7 | Hypoechoic | Uneven | clear | Unclear | 24 |
| 100 | 4.11 | 0.08 | 52.7 | Hypoechoic | even | clear | clear | 6 |
| 101 | 8.01 | 0.15 | 54.4 | Other echo | Uneven | clear | clear | 9 |
| 102 | 6.15 | 0.11 | 57.3 | Other echo | Uneven | Unclear | clear | 15 |
| 103 | 8.64 | 0.12 | 69.4 | Hypoechoic | even | Unclear | clear | 15 |
| 104 | 11.20 | 0.14 | 82.6 | Other echo | even | Unclear | clear | 20 |
| 105 | 20.12 | 0.17 | 115.6 | Hypoechoic | even | Unclear | clear | 10 |
| 106 | 12.30 | 0.16 | 76.2 | Hypoechoic | even | Unclear | clear | 14 |
| 107 | - | - | - | Hypoechoic | even | Unclear | clear | 11 |
| 108 | 0.58 | 0.02 | 23.5 | Hypoechoic | even | Unclear | clear | 7 |
| 109 | 2.13 | 0.06 | 35.9 | Hypoechoic | even | Unclear | clear | 6 |
| 110 | 1.21 | 0.04 | 32.6 | Hypoechoic | even | Unclear | clear | 6 |
| 111 | 0.65 | 0.02 | 35.2 | Hypoechoic | even | Unclear | clear | 4 |
| 112 | 22.60 | 0.46 | 48.8 | Hypoechoic | Uneven | Unclear | clear | 5 |
| 113 | 5.15 | 0.02 | 209.3 | Other echo | even | Unclear | clear | 14 |
| 114 | 10.69 | 0.22 | 48.8 | Hypoechoic | Uneven | Unclear | clear | 13 |
| 115 | 10.18 | 0.17 | 59.1 | Hypoechoic | Uneven | Unclear | clear | 9 |
| 116 | 32.67 | 0.25 | 129.1 | Hypoechoic | even | Unclear | clear | 11 |
| 117 | 14.75 | 0.23 | 64.0 | Hypoechoic | even | Unclear | clear | 9 |
| 118 | 21.33 | 0.48 | 44.7 | Hypoechoic | even | Unclear | clear | 11 |
| 119 | 5.74 | 0.16 | 35.9 | Hypoechoic | Uneven | Unclear | Unclear | 12 |
| 120 | - | - | - | Hypoechoic | even | Unclear | clear | 7 |
| 121 | - | - | - | Hypoechoic | even | Unclear | clear | 13 |
| 122 | - | - | - | Hypoechoic | Uneven | Unclear | clear | 10 |
| 123 | 7.88 | 0.07 | 115.6 | Hypoechoic | even | Unclear | clear | 9 |
| 124 | 30.30 | 0.26 | 118.7 | Hypoechoic | even | Unclear | clear | 24 |

Continued:

| CDUS | CEUS | SE | TNM | PI-RADS V2 | Pathology | Gleason score |
| --- | --- | --- | --- | --- | --- | --- |
| III | Other patterns | 3.21 | T2N0M0 | 4 | PCa | 4 + 3 = 7 |
| I | Other patterns | 5.27 | T2N0M0 | 4 | PCa | 4 + 3 = 7 |
| III | Other patterns | 5.62 | T2N0M0 | 4 | PCa | 3 + 4 = 7 |
| I | Other patterns | 1.94 | T2N0M0 | 5 | PCa | 3 + 4 = 7 |
| III | Other patterns | 1.24 | T2N0M0 | 4 | PCa | 3 + 4 = 7 |
| I | Other patterns | 4.70 | T2N0M0 | 5 | PCa | 3 + 4 = 7 |
| II | Other patterns | 4.53 | T2N0M0 | 4 | PCa | 4 + 3 = 7 |
| III | Other patterns | 2.36 | T2N0M0 | 4 | PCa | 3 + 3 = 6 |
| III | Synchronous wash-in or out, equal enhancement | 0.97 | T2N0M0 | 4 | PCa | 3 + 3 = 6 |
| II | Other patterns | 3.70 | T3N0M0 | 5 | PCa | 4 + 4 = 8 |
| III | Other patterns | 3.86 | T3N0M0 | 5 | PCa | 4 + 3 = 7 |
| I | Other patterns | 1.03 | T3N1M0 | 5 | PCa | 4 + 5 = 9 |
| III | Other patterns | 1.00 | T3N0M0 | 5 | PCa | 4 + 3 = 7 |
| II | Other patterns | 22.90 | T2N0M0 | 5 | PCa | 4 + 4 = 8 |
| II | Other patterns | 4.69 | T2N0M0 | 4 | PCa | 3 + 4 = 7 |
| II | Other patterns | 9.03 | T2N0M0 | 5 | PCa | 4 + 3 = 7 |
| III | Other patterns | 8.55 | T2N0M0 | 4 | PCa | 4 + 4 = 8 |
| II | Other patterns | 0.91 | T2N0M0 | 4 | PCa | 4 + 4 = 8 |
| I | Other patterns | 3.40 | T2N0M0 | 5 | PCa | 3 + 3 = 6 |
| 0 | Synchronous wash-in or out, equal enhancement | 1.01 | T2N0M0 | 3 | PCa | 3 + 3 = 6 |
| III | Other patterns | 5.01 | T3N0M0 | 5 | PCa | 4 + 4 = 8 |
| II | Other patterns | 6.75 | T2N0M0 | 5 | PCa | 4 + 4 = 8 |
| III | Other patterns | 3.68 | T2N0M0 | 5 | PCa | 4 + 4 = 8 |
| III | Other patterns | 5.46 | T2N0M0 | 5 | PCa | 4 + 4 = 8 |
| II | Other patterns | 2.30 | T2N0M0 | 4 | PCa | 4 + 3 = 7 |
| 0 | Synchronous wash-in or out, equal enhancement | 3.86 | T2N0M0 | 4 | PCa | 3 + 4 = 7 |
| I | Other patterns | 5.37 | T2N0M0 | 4 | PCa | 4 + 3 = 7 |
| II | Other patterns | 5.60 | T2N0M0 | 5 | PCa | 4 + 3 = 7 |
| III | Other patterns | 4.64 | T2N0M0 | 5 | PCa | 4 + 3 = 7 |
| III | Other patterns | 6.59 | T2N0M0 | 3 | PCa | 4 + 4 = 8 |
| I | Other patterns | 1.17 | T2N0M0 | 3 | PCa | 3 + 3 = 6 |
| III | Other patterns | 5.99 | T2N0M0 | 4 | PCa | 3 + 4 = 7 |
| III | Other patterns | 27.00 | T2N1M0 | 4 | PCa | 4 + 3 = 7 |
| III | Other patterns | 1.01 | T2N0M0 | 3 | PCa | 3 + 3 = 6 |
| III | Other patterns | 4.92 | T2N0M0 | 5 | PCa | 3 + 4 = 7 |
| III | Other patterns | 1.99 | T2N1M1 | 5 | PCa | 4 + 4 = 8 |
| III | Synchronous wash-in or out, equal enhancement | 3.42 | T2N0M0 | 3 | PCa | 4 + 3 = 7 |
| III | Other patterns | 4.14 | T2N0M0 | 4 | PCa | 4 + 4 = 8 |
| I | Other patterns | 4.82 | T2N0M0 | 4 | PCa | 3 + 3 = 6 |
| III | Other patterns | 10.70 | T2N0M0 | 5 | PCa | 5 + 5 = 10 |
| III | Other patterns | 9.47 | T3N0M0 | 4 | PCa | 3 + 3 = 6 |
| III | Other patterns | 35.00 | T2N0M0 | 5 | PCa | 3 + 3 = 6 |
| III | Other patterns | 2.50 | T2N0M0 | 4 | PCa | 5 + 5 = 10 |
| I | Other patterns | 7.49 | T2N0M0 | 5 | PCa | 3 + 4 = 7 |
| III | Other patterns | 3.49 | T2N0M0 | 4 | PCa | 3 + 3 = 6 |
| II | Other patterns | 3.51 | T2N0M0 | 4 | PCa | 3 + 3 = 6 |
| III | Other patterns | 7.18 | T2N0M0 | 4 | PCa | 4 + 3 = 7 |
| III | Other patterns | 17.57 | T2N0M0 | 4 | PCa | 4 + 5 = 9 |
| III | Other patterns | 4.07 | T2N0M0 | 5 | PCa | 5 + 5 = 10 |
| III | Synchronous wash-in or out, equal enhancement | 8.94 | T2N0M0 | 4 | PCa | 3 + 3 = 6 |
| II | Other patterns | 6.26 | T2N0M0 | 5 | PCa | 4 + 4 = 8 |
| II | Other patterns | 3.80 | T2N0M0 | 5 | PCa | 4 + 3 = 7 |
| I | Other patterns | 1.01 | T2N0M0 | 4 | PCa | 3 + 3 = 6 |
| I | Other patterns | 17.03 | T2N0M0 | 4 | PCa | 3 + 3 = 6 |
| III | Other patterns | 30.84 | T2N0M0 | 4 | PCa | 4 + 5 = 9 |
| III | Other patterns | 14.05 | T3N0M0 | 5 | PCa | 5 + 4 = 9 |
| II | Other patterns | 2.75 | T2N0M0 | 4 | PCa | 3 + 3 = 6 |
| II | Other patterns | 3.56 | T2N0M0 | 4 | PCa | 4 + 4 = 8 |
| III | Synchronous wash-in or out, equal enhancement | 6.10 | T2N0M0 | 4 | PCa | 3 + 3 = 6 |
| II | Other patterns | 30.53 | T2N0M0 | 5 | PCa | 4 + 4 = 8 |
| III | Other patterns | 9.36 | T2N0M0 | 4 | PCa | 4 + 4 = 8 |
| I | Other patterns | 1.87 | T2N0M0 | 5 | PCa | 3 + 4 = 7 |
| III | Other patterns | 5.69 | T2N1M0 | 4 | PCa | 3 + 4 = 7 |
| III | Other patterns | 25.73 | T2N0M0 | 5 | PCa | 4 + 4 = 8 |
| II | Other patterns | 4.69 | T2N1M0 | 5 | PCa | 5 + 5 = 10 |
| II | Other patterns | 4.96 | T2N0M0 | 4 | PCa | 4 + 3 = 7 |
| III | Synchronous wash-in or out, equal enhancement | 28.41 | T3N0M0 | 5 | PCa | 5 + 4 = 9 |
| II | Other patterns | 4.29 | T2N0M0 | 4 | PCa | 4 + 3 = 7 |
| II | Other patterns | 3.20 | T2N0M0 | 5 | PCa | 4 + 4 = 8 |
| I | Other patterns | 2.69 | T2N0M0 | 4 | PCa | 3 + 4 = 7 |
| III | Other patterns | 30.61 | T2N0M1 | 4 | PCa | 4 + 4 = 8 |
| III | Synchronous wash-in or out, equal enhancement | 0.96 | NA | 3 | Non-PCa | NA |
| I | Synchronous wash-in or out, equal enhancement | 5.94 | NA | 3 | Non-PCa | NA |
| I | Synchronous wash-in or out, equal enhancement | 1.07 | NA | 3 | Non-PCa | NA |
| III | Other patterns | 5.12 | NA | 3 | Non-PCa | NA |
| 0 | Synchronous wash-in or out, equal enhancement | 1.08 | NA | 3 | Non-PCa | NA |
| II | Synchronous wash-in or out, equal enhancement | 1.08 | NA | 2 | Non-PCa | NA |
| III | Synchronous wash-in or out, equal enhancement | 1.07 | NA | 2 | Non-PCa | NA |
| 0 | Synchronous wash-in or out, equal enhancement | 1.00 | NA | 3 | Non-PCa | NA |
| III | Other patterns | 1.26 | NA | 3 | Non-PCa | NA |
| 0 | Synchronous wash-in or out, equal enhancement | 1.51 | NA | 3 | Non-PCa | NA |
| I | Other patterns | 1.10 | NA | 3 | Non-PCa | NA |
| II | Other patterns | 1.05 | NA | 2 | Non-PCa | NA |
| 0 | Synchronous wash-in or out, equal enhancement | 1.22 | NA | 2 | Non-PCa | NA |
| I | Other patterns | 1.36 | NA | 2 | Non-PCa | NA |
| I | Synchronous wash-in or out, equal enhancement | 0.84 | NA | 2 | Non-PCa | NA |
| 0 | Other patterns | 1.07 | NA | 3 | Non-PCa | NA |
| I | Synchronous wash-in or out, equal enhancement | 0.50 | NA | 2 | Non-PCa | NA |
| 0 | Other patterns | 1.18 | NA | 2 | Non-PCa | NA |
| I | Synchronous wash-in or out, equal enhancement | 0.92 | NA | 3 | Non-PCa | NA |
| III | Synchronous wash-in or out, equal enhancement | 0.94 | T2N0M0 | 5 | Non-PCa | NA |
| I | Synchronous wash-in or out, equal enhancement | 1.02 | NA | 3 | Non-PCa | NA |
| III | Other patterns | 0.91 | T2N0M0 | 5 | Non-PCa | NA |
| I | Other patterns | 0.87 | NA | 3 | Non-PCa | NA |
| III | Synchronous wash-in or out, equal enhancement | 2.65 | NA | 2 | Non-PCa | NA |
| I | Synchronous wash-in or out, equal enhancement | 1.15 | NA | 2 | Non-PCa | NA |
| I | Synchronous wash-in or out, equal enhancement | 13.80 | NA | 2 | Non-PCa | NA |
| III | Synchronous wash-in or out, equal enhancement | 0.81 | NA | 2 | Non-PCa | NA |
| III | Other patterns | 2.60 | NA | 2 | Non-PCa | NA |
| I | Synchronous wash-in or out, equal enhancement | 0.86 | NA | 3 | Non-PCa | NA |
| I | Synchronous wash-in or out, equal enhancement | 0.85 | NA | 2 | Non-PCa | NA |
| I | Synchronous wash-in or out, equal enhancement | 3.50 | NA | 2 | Non-PCa | NA |
| I | Synchronous wash-in or out, equal enhancement | 3.85 | NA | 2 | Non-PCa | NA |
| I | Synchronous wash-in or out, equal enhancement | 5.05 | NA | 2 | Non-PCa | NA |
| I | Synchronous wash-in or out, equal enhancement | 0.72 | NA | 2 | Non-PCa | NA |
| I | Other patterns | 0.75 | T2N0M0 | 4 | Non-PCa | NA |
| 0 | Synchronous wash-in or out, equal enhancement | 0.98 | NA | 2 | Non-PCa | NA |
| I | Synchronous wash-in or out, equal enhancement | 1.03 | NA | 2 | Non-PCa | NA |
| II | Synchronous wash-in or out, equal enhancement | 1.43 | NA | 3 | Non-PCa | NA |
| 0 | Other patterns | 1.02 | T2N0M0 | 4 | Non-PCa | NA |
| I | Synchronous wash-in or out, equal enhancement | 1.11 | NA | 2 | Non-PCa | NA |
| I | Synchronous wash-in or out, equal enhancement | 0.93 | NA | 2 | Non-PCa | NA |
| I | Synchronous wash-in or out, equal enhancement | 10.56 | NA | 2 | Non-PCa | NA |
| III | Synchronous wash-in or out, equal enhancement | 6.20 | T2N0M0 | 5 | Non-PCa | NA |
| III | Synchronous wash-in or out, equal enhancement | 0.91 | NA | 2 | Non-PCa | NA |
| I | Synchronous wash-in or out, equal enhancement | 0.88 | NA | 2 | Non-PCa | NA |
| I | Other patterns | 1.02 | T2N0M0 | 5 | Non-PCa | NA |
| I | Synchronous wash-in or out, equal enhancement | 0.86 | NA | 3 | Non-PCa | NA |
| III | Synchronous wash-in or out, equal enhancement | 2.84 | NA | 3 | Non-PCa | NA |
| I | Other patterns | 1.11 | T2N0M0 | 5 | Non-PCa | NA |
| I | Synchronous wash-in or out, equal enhancement | 24.80 | T2N0M0 | 4 | Non-PCa | NA |
| 0 | Synchronous wash-in or out, equal enhancement | 1.06 | NA | 3 | Non-PCa | NA |
| I | Synchronous wash-in or out, equal enhancement | 1.10 | NA | 2 | Non-PCa | NA |
| III | Synchronous wash-in or out, equal enhancement | 0.90 | NA | 2 | Non-PCa | NA |

(“-” refers to data from a second nodule in the same patient.)

PSA: Prostate-specific antigen; PSAD: Prostate-specific antigen density; PV: Prostate volume; CDUS: Color Doppler ultrasound; CEUS: Contrast-enhanced ultrasound; SE: Strain elastography; PI-RADS V2: Prostate imaging reporting and data system version 2; NA: Not available
