# Supplementary material for: Multiparametric transrectal ultrasound for the diagnosis of peripheral zone prostate cancer and clinically significant prostate cancer: novel scoring systems
Source: BMC Urol. 2022 Apr 19;22:64. doi: 10.1186/s12894-022-01013-8 (PMC9016931; doi:10.1186/s12894-022-01013-8)
Supplement: Supplementary file 2 — Additional file 2. Lesions variables in the validation cohort, shows all TRUS parameters and preoperative variables for each lesion in the validation cohort. [file 12894_2022_1013_MOESM2_ESM.docx]

Additional file 2. Lesions variables in the validation cohort.

| Lesions  number | PSA  (ng/mL) | PSAD  (ng/mL/cm^3^) | PV  (mL) | echogenicity | distribution | margin | demarcation of internal and external glands | Size  (mm) |
| --- | --- | --- | --- | --- | --- | --- | --- | --- |
| 1 | 7.12 | 0.06 | 117.8 | Hypoechoic | Uneven | Unclear | Clear | 11 |
| 2 | 16.70 | 0.73 | 23.0 | Hypoechoic | Uneven | Unclear | Clear | 10 |
| 3 | 6.59 | 0.17 | 37.7 | Hypoechoic | Even | Unclear | Clear | 11 |
| 4 | 10.50 | 0.20 | 52.7 | Hypoechoic | Even | Unclear | Clear | 12 |
| 5 | - | - | - | Hypoechoic | Even | Unclear | Clear | 9 |
| 6 | 10.24 | 0.17 | 61.6 | Hypoechoic | Uneven | Unclear | Unclear | 7 |
| 7 | - | - | - | Hypoechoic | Uneven | Unclear | Unclear | 14 |
| 8 | 12.00 | 0.21 | 57.7 | Hypoechoic | Even | Clear | Unclear | 22 |
| 9 | 18.12 | 0.45 | 40.6 | Hypoechoic | Even | Unclear | Clear | 12 |
| 10 | - | - | - | Hypoechoic | Uneven | Unclear | Clear | 7.1 |
| 11 | 9.89 | 0.19 | 52.0 | Hypoechoic | Even | Unclear | Unclear | 16 |
| 12 | 9.23 | 0.33 | 28.4 | Hypoechoic | Even | Clear | Unclear | 8.6 |
| 13 | 10.20 | 0.17 | 59.6 | Hypoechoic | Uneven | Unclear | Clear | 10 |
| 14 | 26.40 | 0.22 | 121.5 | Hypoechoic | Uneven | Unclear | Clear | 16 |
| 15 | 17.20 | 0.18 | 93.7 | Other echo | Uneven | Unclear | Unclear | 40 |
| 16 | 3.84 | 0.14 | 27.2 | Hypoechoic | Even | Unclear | Clear | 14 |
| 17 | 15.50 | 0.54 | 28.6 | Hypoechoic | Even | Unclear | Unclear | 11 |
| 18 | 7.82 | 0.17 | 45.4 | Hypoechoic | Uneven | Unclear | Clear | 11 |
| 19 | 14.40 | 0.47 | 30.8 | Hypoechoic | Uneven | Clear | Clear | 15 |
| 20 | 23.70 | 0.74 | 32.2 | Hypoechoic | Uneven | Unclear | Clear | 16 |
|  | 14.30 | 0.45 | 31.8 | Hypoechoic | Even | Unclear | Clear | 15 |
|  | 14.10 | 0.38 | 37.1 | Hypoechoic | Even | Unclear | Unclear | 8 |
|  | 18.60 | 0.36 | 51.9 | Hypoechoic | Even | Unclear | Clear | 12 |
|  | 9.42 | 0.25 | 37.6 | Hypoechoic | Uneven | Unclear | Unclear | 19 |
|  | 3.67 | 0.05 | 69.7 | Hypoechoic | Uneven | Clear | Clear | 5 |
|  | - | - | - | Hypoechoic | Even | Unclear | Clear | 8 |
|  | 16.00 | 0.28 | 56.5 | Hypoechoic | Even | Unclear | Unclear | 14 |
|  | ＞100 | 2.76 | 36.2 | Hypoechoic | Even | Unclear | Clear | 20 |
|  | 48.80 | 1.24 | 39.4 | Hypoechoic | Uneven | Unclear | Clear | 8 |
| 30 | 81.20 | 0.83 | 97.6 | Hypoechoic | Even | Unclear | Unclear | 12 |
| 31 | 6.51 | 0.21 | 30.8 | Hypoechoic | Uneven | Unclear | Unclear | 20 |
| 32 | 8.89 | 0.19 | 47.7 | Hypoechoic | Uneven | Unclear | Unclear | 7 |
| 33 | 6.54 | 0.09 | 71.4 | Hypoechoic | Even | Unclear | Clear | 10 |
| 34 | 2.68 | 0.07 | 39.5 | Hypoechoic | Even | Unclear | Clear | 7 |
| 35 | ＞100 | 1.57 | 63.7 | Hypoechoic | Even | Unclear | Unclear | 23 |
| 36 | 5.02 | 0.11 | 45.3 | Hypoechoic | Uneven | Clear | Clear | 7 |
| 37 | 9.40 | 0.24 | 38.9 | Hypoechoic | Uneven | Clear | Clear | 10 |
| 38 | ＞100 | 1.59 | 63.0 | Hypoechoic | Even | Unclear | Unclear | 26 |
| 39 | 10.60 | 0.34 | 31.6 | Hypoechoic | Uneven | Unclear | Unclear | 15 |
| 40 | 24.70 | 0.38 | 64.3 | Hypoechoic | Even | Unclear | Unclear | 17 |
| 41 | ＞100 | 2.40 | 41.6 | Hypoechoic | Uneven | Unclear | Clear | 24 |
| 42 | ＞100 | 2.46 | 40.6 | Hypoechoic | Even | Unclear | Unclear | 18 |
| 43 | ＞100 | 1.32 | 75.8 | Hypoechoic | Even | Unclear | Unclear | 21 |
| 44 | 6.93 | 0.13 | 52.6 | Other echo | Uneven | Clear | Clear | 9 |
| 45 | 61.70 | 0.73 | 84.6 | Hypoechoic | Uneven | Unclear | Unclear | 17 |
| 46 | 7.31 | 0.28 | 26.5 | Hypoechoic | Even | Clear | Unclear | 5 |
| 47 | 4.27 | 0.09 | 49.1 | Hypoechoic | Even | Clear | Clear | 22 |
| 48 | 4.73 | 0.10 | 46.4 | Hypoechoic | Even | Clear | Clear | 4 |
| 49 | 7.52 | 0.05 | 154.2 | Hypoechoic | Even | Clear | Clear | 8 |
| 50 | 4.23 | 0.04 | 111.0 | Hypoechoic | Uneven | Clear | Clear | 18 |
| 51 | 4.26 | 0.06 | 70.8 | Hypoechoic | Even | Clear | Clear | 7 |
| 52 | 9.92 | 0.20 | 48.7 | Hypoechoic | Even | Unclear | Unclear | 12 |
| 53 | 1.09 | 0.04 | 26.1 | Hypoechoic | Even | Clear | Clear | 9 |
| 54 | 10.70 | 0.17 | 63.7 | Hypoechoic | Even | Clear | Clear | 11 |
| 55 | 4.87 | 0.05 | 95.5 | Hypoechoic | Even | Clear | Clear | 8 |
| 56 | 14.20 | 0.18 | 79.5 | Hypoechoic | Uneven | Unclear | Clear | 14 |
| 57 | - | - | - | Other echo | Even | Clear | Clear | 17 |
| 58 | 1.51 | 0.02 | 100.2 | Hypoechoic | Even | Clear | Clear | 31 |
| 59 | 2.77 | 0.05 | 54.4 | Hypoechoic | Even | Clear | Clear | 12 |
| 60 | 15.26 | 0.22 | 68.6 | Hypoechoic | Even | Unclear | Unclear | 10 |
| 61 | ＞100 | 1.60 | 62.4 | Hypoechoic | Even | Clear | Clear | 9 |
| 62 | 17.60 | 0.20 | 86.0 | Hypoechoic | Uneven | Clear | Clear | 16 |
| 63 | 14.90 | 0.16 | 91.8 | Hypoechoic | Even | Clear | Clear | 9 |
| 64 | 4.60 | 0.08 | 58.1 | Hypoechoic | Even | Clear | Clear | 12 |
| 65 | 20.30 | 0.35 | 57.2 | Hypoechoic | Even | Clear | Unclear | 7 |
| 66 | 5.75 | 0.09 | 64.3 | Hypoechoic | Even | Clear | Unclear | 12 |
| 67 | 7.95 | 0.14 | 56.1 | Hypoechoic | Even | Clear | Unclear | 8 |
| 68 | 6.38 | 0.14 | 45.0 | Hypoechoic | Uneven | Clear | Clear | 6 |
| 69 | 20.20 | 0.16 | 128.4 | Hypoechoic | Even | Clear | Clear | 10 |
| 70 | 5.57 | 0.12 | 47.7 | Hypoechoic | Even | Unclear | Unclear | 10 |
| 71 | 14.41 | 0.34 | 42.4 | Hypoechoic | Even | Clear | Clear | 13 |
| 72 | 3.43 | 0.04 | 76.7 | Hypoechoic | Even | Clear | Unclear | 27 |

Continued:

| CDUS | CEUS | | SE | TNM | PI-RADS V2 | Pathology | Gleason score |
| --- | --- | --- | --- | --- | --- | --- | --- |
| III | | Other patterns | 4.49 | T2N0M0 | 4 | PCa | 4 + 3 = 7 |
| I | | Other patterns | 2.31 | T2N0M0 | 5 | PCa | 4 + 3 = 7 |
| III | | Other patterns | 5.27 | T2N0M0 | 4 | PCa | 4 + 4 = 8 |
| II | | Other patterns | 4.53 | T2N0M0 | 4 | PCa | 3 + 4 = 7 |
| III | | Other patterns | 3.56 | T2N0M0 | 4 | PCa | 3 + 4 = 7 |
| III | | Other patterns | 1.14 | T2N0M0 | 5 | PCa | 4 + 3 = 7 |
| III | | Other patterns | 2.81 | T2N0M0 | 4 | PCa | 3 + 3 = 6 |
| I | | Synchronous wash-in or out, equal enhancement | 5.48 | T2N0M0 | 5 | PCa | 3 + 4 = 7 |
| III | | Other patterns | 1.93 | T2N0M0 | 4 | PCa | 4 + 3 = 7 |
| III | | Other patterns | 3.66 | T2N0M0 | 4 | PCa | 4 + 3 = 7 |
| III | | Other patterns | 4.29 | T2N0M0 | 5 | PCa | 4 + 3 = 7 |
| III | | Other patterns | 2.02 | T2N0M0 | 5 | PCa | 4 + 3 = 7 |
| II | | Other patterns | 20.10 | T2N0M0 | 4 | PCa | 3 + 3 = 6 |
| III | | Other patterns | 19.40 | T2N0M0 | 5 | PCa | 4 + 5 = 9 |
| II | | Other patterns | 5.46 | T3N1M0 | 5 | PCa | 4 + 4 = 8 |
| II | | Other patterns | 5.65 | T2N0M0 | 4 | PCa | 3 + 4 = 7 |
| II | | Other patterns | 3.10 | T2N0M0 | 5 | PCa | 5 + 5 = 10 |
| III | | Other patterns | 1.21 | T2N0M0 | 4 | PCa | 3 + 4 = 7 |
| III | | Other patterns | 4.00 | T2N0M0 | 4 | PCa | 4 + 3 = 7 |
| III | | Other patterns | 7.25 | T2N0M0 | 5 | PCa | 4 + 3 = 7 |
| I | | Other patterns | 3.01 | T2N0M0 | 4 | PCa | 4 + 4 = 8 |
| I | | Other patterns | 1.87 | T2N0M0 | 4 | PCa | 3 + 4 = 7 |
| II | | Other patterns | 1.64 | T2N0M0 | 4 | PCa | 4 + 3 = 7 |
| II | | Other patterns | 21.40 | T2N0M0 | 4 | PCa | 3 + 4 = 7 |
| II | | Other patterns | 23.80 | T2N0M0 | 4 | PCa | 3 + 3 = 6 |
| I | | Other patterns | 1.70 | T2N0M0 | 2 | PCa | 3 + 3 = 6 |
| III | | Other patterns | 1.82 | T2N0M0 | 3 | PCa | 3 + 4 = 7 |
| III | | Other patterns | 6.77 | T3N0M1 | 5 | PCa | 5 + 4 = 9 |
| I | | Other patterns | 1.85 | T2N0M0 | 5 | PCa | 4 + 3 = 7 |
| III | | Other patterns | 2.41 | T3N0M0 | 5 | PCa | 4 + 4 = 8 |
| II | | Other patterns | 5.97 | T2N0M0 | 5 | PCa | 5 + 5 = 10 |
| I | | Other patterns | 5.11 | T2N0M0 | 4 | PCa | 4 + 3 = 7 |
| II | | Other patterns | 1.52 | T2N0M0 | 4 | PCa | 3 + 3 = 6 |
| II | | Other patterns | 4.30 | T2N0M0 | 4 | PCa | 3 + 3 = 6 |
| II | | Other patterns | 1.85 | T4N0M0 | 5 | PCa | 5 + 4 = 9 |
| 0 | | Synchronous wash-in or out, equal enhancement | 1.24 | T2N0M0 | 4 | PCa | 3 + 4 = 7 |
| II | | Synchronous wash-in or out, equal enhancement | 3.02 | T2N0M0 | 4 | PCa | 3 + 4 = 7 |
| III | | Other patterns | 3.46 | T3N0M1 | 5 | PCa | 4 + 4 = 8 |
| III | | Other patterns | 4.03 | T2N0M0 | 4 | PCa | 3 + 4 = 7 |
| III | | Other patterns | 2.20 | T3N1M0 | 5 | PCa | 5 + 4 = 9 |
| III | | Other patterns | 2.79 | T3N0M1 | 5 | PCa | 4 + 5 = 9 |
| III | | Other patterns | 8.69 | T3N1M0 | 5 | PCa | 4 + 5 = 9 |
| III | | Other patterns | 6.77 | T3N1M1 | 5 | PCa | 5 + 4 = 9 |
| I | | Synchronous wash-in or out, equal enhancement | 3.69 | NA | 3 | Non-PCa | NA |
| II | | Other patterns | 1.66 | T2N1M0 | 5 | Non-PCa | NA |
| 0 | | Synchronous wash-in or out, equal enhancement | 5.82 | T2N0M0 | 4 | Non-PCa | NA |
| III | | Synchronous wash-in or out, equal enhancement | 0.70 | NA | 2 | Non-PCa | NA |
| 0 | | Synchronous wash-in or out, equal enhancement | 1.01 | NA | 3 | Non-PCa | NA |
| 0 | | Synchronous wash-in or out, equal enhancement | 1.15 | NA | 2 | Non-PCa | NA |
| I | | Synchronous wash-in or out, equal enhancement | 3.31 | NA | 2 | Non-PCa | NA |
| I | | Synchronous wash-in or out, equal enhancement | 0.85 | NA | 2 | Non-PCa | NA |
| II | | Other patterns | 1.57 | NA | 3 | Non-PCa | NA |
| I | | Synchronous wash-in or out, equal enhancement | 2.60 | NA | 2 | Non-PCa | NA |
| I | | Other patterns | 1.15 | NA | 2 | Non-PCa | NA |
| 0 | | Synchronous wash-in or out, equal enhancement | 1.28 | T2N0M0 | 4 | Non-PCa | NA |
| I | | Synchronous wash-in or out, equal enhancement | 1.67 | NA | 3 | Non-PCa | NA |
| I | | Synchronous wash-in or out, equal enhancement | 0.71 | NA | 3 | Non-PCa | NA |
| III | | Synchronous wash-in or out, equal enhancement | 1.13 | NA | 2 | Non-PCa | NA |
| II | | Synchronous wash-in or out, equal enhancement | 1.34 | NA | 2 | Non-PCa | NA |
| 0 | | Synchronous wash-in or out, equal enhancement | 1.10 | NA | 3 | Non-PCa | NA |
| I | | Synchronous wash-in or out, equal enhancement | 1.63 | NA | 2 | Non-PCa | NA |
| II | | Synchronous wash-in or out, equal enhancement | 0.74 | NA | 3 | Non-PCa | NA |
| I | | Other patterns | 7.77 | NA | 3 | Non-PCa | NA |
| II | | Synchronous wash-in or out, equal enhancement | 0.76 | T2N0M0 | 4 | Non-PCa | NA |
| 0 | | Synchronous wash-in or out, equal enhancement | 0.94 | NA | 3 | Non-PCa | NA |
| 0 | | Synchronous wash-in or out, equal enhancement | 0.87 | NA | 2 | Non-PCa | NA |
| I | | Synchronous wash-in or out, equal enhancement | 0.98 | NA | 2 | Non-PCa | NA |
| 0 | | Synchronous wash-in or out, equal enhancement | 1.66 | NA | 2 | Non-PCa | NA |
| 0 | | Other patterns | 1.56 | T2N0M0 | 4 | Non-PCa | NA |
| III | | Other patterns | 4.29 | T2N0M0 | 4 | Non-PCa | NA |
| I | | Synchronous wash-in or out, equal enhancement | 1.25 | NA | 2 | Non-PCa | NA |
| III | | Synchronous wash-in or out, equal enhancement | 0.34 | T2N0M0 | 2 | Non-PCa | NA |

(“-” refers to data from a second nodule in the same patient.)

PSA: Prostate-specific antigen; PSAD: Prostate-specific antigen density; PV: Prostate volume; CDUS: Color Doppler ultrasound; CEUS: Contrast-enhanced ultrasound; SE: Strain elastography; PI-RADS V2: Prostate imaging reporting and data system version 2
